# *In vitro* effect of recombinant amaranth cystatin (AhCPI) on spore germination, mycelial growth, stress response and cellular integrity of *Aspergillus niger* and *Aspergillus parasiticus*

**DOI:** 10.1080/21501203.2015.1112857

**Published:** 2015-11-19

**Authors:** Dora Linda Guzmán-de-Peña, Ana María Correa-González, Laura Valdés-Santiago, Claudia G. León-Ramírez, Silvia Valdés-Rodríguez

**Affiliations:** a Departamento de Biotecnología y Bioquímica, Unidad Irapuato Centro de Investigación y Estudios Avanzados-IPN, Km 9.6 Libramiento Norte Irapuato-León, 36821Irapuato, Guanajuato, México; b Departamento de Ingeniería Genética, Unidad Irapuato Centro de Investigación y Estudios Avanzados-IPN, Km 9.6 Libramiento Norte Irapuato-León, 36821Irapuato, Guanajuato, México

**Keywords:** cystatin, *Aspergillus niger*, *Aspergillus parasiticus*, antifungal activity, stress response, growth inhibition

## Abstract

The inhibitory effect of recombinant amaranth cystatin (AhCPI) on the spore germination and growth of the mycotoxigenic fungus *Aspergillus parasiticus* and *Aspergillus niger* was investigated. AhCPI showed a concentration-dependent antifungal activity against both fungi. Differential effects were observed when fungi were treated with cystatin in two developmental stages. When AhCPI was added to young mycelium cultures of *A. niger*, it had a dramatic effect on mycelial growth compared with old mycelium cultures. On the contrary, there was no differential effect of AhCPI addition to either old or young mycelium of *A. parasiticus*. Furthermore, electron microscopic observations showed that cystatin caused important effects at the level of cell morphology and organelle integrity of both fungi. Additionally, *A. parasiticus* spores treated with AhCPI presented sensitivity to oxidative, osmotic and ionic stresses; in opposition, under same conditions, *A. niger* did not show sensitivity to any stressful agent. These results suggest that AhCPI antifungal activity might be related with damage to cell integrity, affecting the survival of the fungi. In addition, our evidences showed that fungal species respond dissimilarly to cystatin; however, such disparities can be used to the control of unwanted fungi.

## 1. Introduction

Phytocystatins are cysteine proteinase inhibitors implied in several physiological processes (Habid & Fazili 2007; Benchabane et al. 2010). They have been related with defence mechanisms against insects and plant pathogens as well as plant response to heat, cold and saline stresses (Pernas et al. 2000; Carrillo et al. 2011; Valdés-Rodríguez et al. 2015). It is well documented that phytocystatins present antifungal activity against a wide range of pathogenic fungi such as *Botritys cinerea, Plectosphaerella cucumerina, Colletotrichum graminicola, Colletotrichum capsisi, Pyricularia grisea, Fusarium oxysporum, Septoria nodorum, Phytophthora nicotianae, Sclerotiana sclerotiorum, Sclerotium rolfsii, Alternaria brassicae, Glomerella cingulata, Pythium aphanidermatum, Rhizoctonia solani, Trichoderma reesei, Aspergillus sydowii, Helminthosporium sesamum* (Pernas et al. 1999; Sugawara et al. 2002; Martínez et al. 2003; Yang & Yeh 2005; Abraham et al. 2006; Porruan et al. 2013; Cheng et al. 2014). However, the mechanism by which phytocystatins inhibit fungal growth is not yet known. Interestingly, plant cystatins do not have antibacterial activity against several species of either Gram-positive or Gram-negative bacteria (Pernas et al. 1999; Yang & Yeh 2005; Cheng et al. 2014).

Previously, we isolated an amaranth cystatin cDNA from developing amaranth seed encoding the recombinant amaranth cystatin (AhCPI). AhCPI was expressed in *Escherichia coli* and purified by nickel affinity chromatography (Valdés-Rodríguez et al. 2007, 2010). This is a 24 kDa protein highly related in sequence to phytocystatin with the C-terminal extension. It was proved that AhCPI was able to inhibit the growth of some phytopathogenic fungi such as *Fusarium oxysporum, Sclerotium cepivorum* and *Rhyzoctonia solani*; however, this effect was not related to its ability to inhibit the activity of mycelial proteases (Valdés-Rodríguez et al. 2010). In agreement, the antifungal effect of barley cystatin (Hv-CPI) and its derived mutants does not correlate with their activities as proteinase inhibitors (Martínez et al. 2003). All of these results are supported by evidences presented by Wang et al., based on the inhibitory kinetics of different segments of tarocystatins on papain activity. They demonstrated that the N-terminal segment of protein showed greater antifungal activity than the full-length protein, indicating that the antifungal effect was not related to proteinase inhibitory activity (Wang et al. 2008). Furthermore, a connection between modifications in fungal membrane permeability and the antifungal properties of the proteinase inhibitors has been suggested (Giudici et al. 2000). Therefore, a study that provides more detailed data about the mode of action of the antifungal cystatin is necessary. In this study, we examined the *in vitro* growth inhibition of *A. parasiticus* (mycotoxigenic fungus) and *A. niger* (generally recognized as safe (GRAS) organism) by AhCPI and attempted to elucidate the possible mechanism of fungal growth inhibition. The antifungal activity against these fungi has not been analysed, even though the mycotoxigenic fungi *A. flavus* and *A. parasiticus* represent a hazard to agricultural production, human health and animal production (Zain 2011). Whereas *A. niger* is one of the fungi most used in the production of fermented food, enzyme and organic acid for human consumption (Schuster et al. 2002). Since cystatins represent a promising tool for diverse applications in the control of fungi, this knowledge could allow the development of strategies to control fungal contamination of food. Some advantages of the use of phytocystatin are: the high antifungal activity, and the low toxicity towards higher organisms.

## 2. Materials and methods

### 2.1. Expression and purification of recombinant cystatin

The isolation of recombinant AhCPI from transformed *E. coli* cells as well as its purification by affinity chromatography was done according to the protocol reported by Valdés-Rodríguez et al. (2010). *Escherichiacoli* M15 (pREP 4) cells containing the amaranth cystatin coding sequence were grown at 37°C until they reached an OD600 of 0.5. The expression of cystatin was induced at 37°C by the addition of 0.1 mM Isopropyl beta-D-1-thiogalactopyranoside, and the cells were harvested by centrifugation at 17,300 × *g* for 10 min after 5 h of incubation. From cell lysates, AhCPI was purified using an affinity nickel resin column, previously equilibrated with phosphate buffer. The cystatin was eluted by 250 mM imidazol dissolved in the same buffer. The purified cystatin was exhaustively dialysed against water in a microdialysis system.

### 2.2. Antifungal assay of AhCPI

The antimicrobial toxicity of AhCPI was assayed with two different types of fungi: *Aspergillus parasiticus*, American Type Culture Collection (ATCC) 16,992 (aflatoxin producer) and *Aspergillus niger* ATCC 1015 (acid organic producer – GRAS) (ATCC, www.atcc.org). Fungal strains were grown in potato dextrose agar (PDA, Difco) for 6 and 4 days, respectively. Conidia were harvested by washing the mycelium colony with 10 mL of water plus Triton X-100 solution (0.01%), and counted with a haemocytometer to adjust to the desired inoculum concentration with sterile distilled water. Before inoculation, conidia suspensions were kept overnight at 4°C to ensure optimal germination. In order to analyse the effect of AhCPI on mycelial growth, spore suspensions (≈1000 viable spores) of *A. niger* and *A. parasiticus* were cultivated on potato dextrose broth (PDB) media at 28°C with addition of different concentrations of AhCPI (1.5, 5, 10, 15, 20, 25 and 30 μM) in 250 μL as total volume in a microtiter plate. After different periods of incubation, fungal growth was measured by absorbance at 492 nm in a microplate spectrometer (Benchmark plus®, Bio-Rad, Hercules, CA, USA). The assays were performed with 12 biological replicates.

### 2.3. Effect of cystatin on fungi in different stages of development

Spore suspensions of each fungus (1 × 10^3^ spores mL^−1^) were grown in microtiter plates in absence of AhCPI under the same conditions described earlier. Spores were allowed to grow for 28 and 43 h before different concentrations (20 µM in the case of *A. niger* and 30 µM in the case of *A. parasiticus*) of the AhCPI were added to the cultures. Microtiter plates were incubated for additional 44 and 29 h, respectively, to reach 72 h in all treatments. Samples from the different conditions were taken for microscopic observations.

### 2.4. Microscopic observations of spore of A. parasiticus and A. niger after AhCPI exposure

Fungal spore suspensions were incubated at 4°C overnight. Afterwards, a spore suspension of each fungus (1 × 10^9^ spores in 60 µl of water plus 0.01% triton) was inoculated separately in 4 ml of PDB and incubated at 28°C. Then AhCPI at final concentration of 10 µM and 20 µM was added to the *A. niger* and *A parasiticus* spore suspensions. No AhCPI was added to the control cultures of both fungi. Flasks containing the treatments were incubated for 65 h at 28°C, and samples were taken for microscopic observations.

### 2.5. Preparation of fixed cells for electron microscopic observations

To investigate the effect of AhCPI on spore morphology, we proved 20 and 30 μM of AhCPI which inhibit germination and mycelial growth for both fungi. Spores of *A. niger* and *A. parasiticus* were incubated in PDB for 18 h at 4°C without cystatin to obtain swollen spores. Afterwards, 20 and 30 μM of AhCPI were added to *A. niger* and *A. parasiticus*, respectively, and the cultures were incubated for 91 h at 28°C. For electron microscopy, the cultures were harvested and pelleted. Samples with paraformaldehyde-glutaraldehyde were fixed and postfixed with osmium tetroxide, as reported by Cabrera-Ponce et al. (2012). A JM-1010 electron microscope was used for observations. Swollen spores without cystatin were fixed and used as control.

### 2.6. Response of spores treated with AhCPI to different types of stress

**Figure 1.**
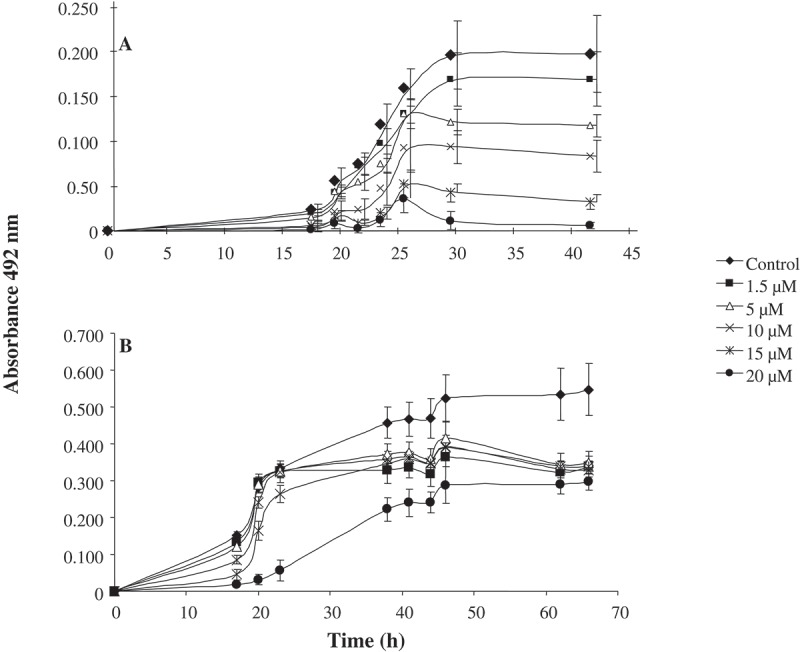
*In vitro* growth curves. Spores (1 × 10^3^) were incubated in 250 μL of PDB with several concentrations of AhCPI. (**A**) *Aspergillus niger* and (**B**) *Aspergillus parasiticus* in the presence of 1.5–20 μM of AhCPI. Fungal growth was measured by changes in optical density at 492 nm at different times. The assays were performed with 12 biological repetitions and values represent the mean ± SD.

Spores (10^6^ spores mL^−1^) of *A. niger* and *A. parasiticus* were incubated for 96 h at 28°C in PDB with 15 and 20 μM of AhCPI, respectively, in microtiter plates. These concentrations of AhCPI were selected because fungal growth was affected at the same level (Figure 1). Spore suspensions were adjusted to 10^5^ spores mL^−1^ (counted with a Neubauer chamber), 10-fold serial dilutions were prepared, and 10 μL of each fungus was spotted on PDA plates with addition of different compounds: 1% H_2_O_2_; 0.05 mM Rose bengal; 30 mM LiCl; 2 M Sorbitol. Fresh spores incubated previously at 4°C overnight and not treated with AhCPI were used as control. Plates were incubated at 28°C for 72 h and photographed with a Cannon camera model Eorebeld.

## 3. Results and discussion

### 3.1. In vitro mycelial growth inhibition of A. niger and A. parasiticus by recombinant AhCPI

The effect of purified AhCPI on the mycelial growth of *A. niger* (Figure 1A) and *A. parasiticus* (Figure 1B) was determined by *in vitro* bioassays. Both fungi presented a dose-dependent effect of AhCPI on mycelial growth. However, the antifungal effect in *A. niger* was stronger, since the growth measured by absorbance at 492 nm was lesser compared with the growth observed in *A. parasiticus*. Five concentrations of AhCPI were used (1.5, 5, 10, 15 and 20 μM), and increase in absorbance was indicative of spore germination and mycelial growth. At higher concentrations of cystatin, there was inhibition of spore germination, since absorbance measurements were lesser (indicating no fungal growth, see Figure 3A) compared with those presented at lower concentrations of AhCPI. We observed that at 20 μM, *A. niger* did not present increase in absorbance (Figure 1A), indicating 100% of growth inhibition. In contrast, the absorbance in cultures of *A. parasiticus* with AhCPI was half of that of the control at 60 h of incubation in all tested concentrations (Figure 1B). In order to confirm the antifungal effect of AhCPI on *A. niger*, the dry weight of mycelial growth after 65 h of incubation with 15 and 20 μM of AhCPI was determined. At 15 μM, there was a reduction of 65% of mycelium dry weight compared with the untreated cells, and at 20 μM there was a reduction of 100% (data not shown). Regarding *A. parasiticus*, the fungus showed higher tolerance to AhCPI treatment than *A. niger*, and higher concentrations of AhCPI were tested. Concentrations of 20 and 25 μM of AhCPI inhibited 23.2% and 61.2% of fungal growth, respectively, while 30 μM inhibited 82.79 % (data not shown). The effective concentration for 50% growth inhibition (EC50) was about 12.75 µM for *A niger* and 21 µM for *A. parasiticus*. It is interesting to observe the wide range of concentrations in which AhCPI affects different fungal species. The reported EC50 in *Fusarium oxysporum* was 13 μM; in *Slerotium cepivorum*, it was 0.38 μM, and in *Rhyzoctonia solani*, it was 0.16 μM (Valdés-Rodríguez et al. 2010). Interestingly *A. niger* and *A. parasiticus* showed an EC50 slightly less than twice, as was determined in this work. These results, when compared with the EC50 reported for other fungi, suggested that the antifungal effect of AhCPI might depend on the fungal species.

**Figure 2.**
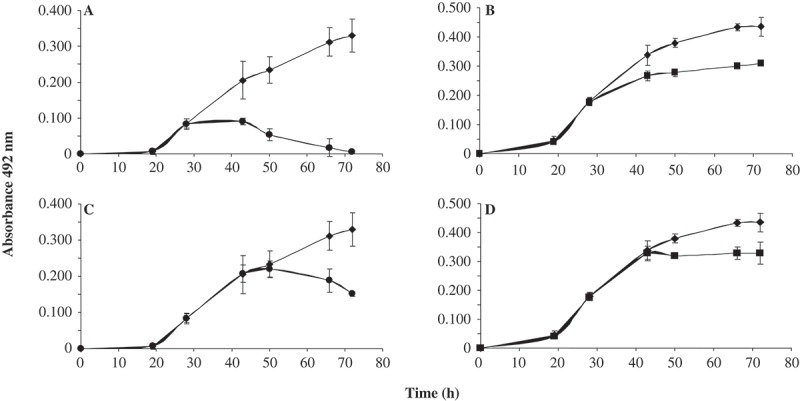
Effect of AhCPI on mycelial growth in two different developmental stages. Spores (1 × 10^3^) of each fungus were grown on AhCPI-free PDB and incubated at 28°C for 28 h and 43 h and then AhCPI was added. (**A**) *A. niger* treated with 20 µM cystatin; (**B**) *A. parasiticus* treated with 30 µM cystatin; (**A**), (**B**) AhCPI was added to young mycelium (28 h); (**C**), (**D**) AhCPI was added to old mycelium (43 h), and both treatments were incubated for 72 h. Control mycelia are represented with black diamond; 20 μM AhCPI *A. niger* treatments are represented with black circles and 30 μM AhCPI *A. parasiticus* treatments are represented with black squares. Fungal growth was measured by changes in optical density at 492 nm. Values indicate means of 12 biological repetitions ±SD.

**Figure 3.**
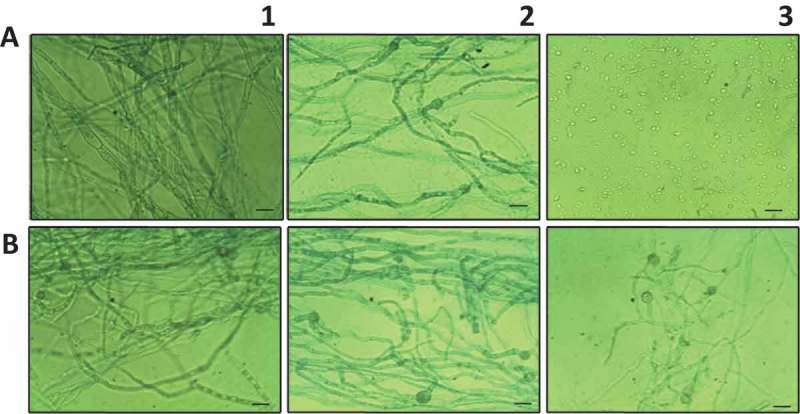
Microscopic observation of the effect of AhCPI on mycelial development. Cystatin was added to spore suspensions and incubated for 65 h before micrographs were taken. (**A**) *A. niger*; (**B**) *A. parasiticus*. (**1**) Spores without cystatin; (**2**) 10 µM AhCPI; (**3**) 20µ M AhCPI. Scale bars = 20 μm.

The antifungal activity of plant cystatins from different sources has been reported previously. Cystatin from pearl millet (*Pennisetum glaucum*) inhibited the mycelial growth of *Claviceps paspali, Claviceps purpurea, Curvularia fallax, Curvularia cymbopogonis, Curvularia lunata*, as well as *Alternaria solani* and *Fusarium oxysporum* (Joshi et al. 1998). Canecystatin affected spore germination of *Trichoderma reesei* (Soares-Costa et al. 2002). The phytocystatin isolated from taro corms (*Colocasia esculenta*) was able to inhibit mycelial growth and cause the lysis of sclerotia in *Sclerotium rofsii* (Yang & Yeh 2005). The recombinant barley and chestnut cystatin inhibited the growth of *Botrytis cinerea, Colletotrichum graminicola, Septoria nodorum* and *Plectosphaerella cucumerina*; interestingly, it did not affect *Trichoderma viride* (Pernas et al. 2000; Martínez et al. 2003). In the case of cystatin from siam tulip, the protein abolished mycelial growth of *F. oxysporum, Colletotrichum capsici* and *Pyricularia grisea* at different levels; 5 μM inhibited almost 100% of *P. grisea* whereas, *F. oxysporum* and *C. capsici* required almost 10 μM to reach the same level of inhibition (Porruan et al. 2013). The differences in sensivity to cystatin between species could be given by developmental stage and/or cell wall composition (see below).

### 3.2. Mycelial growth of A. niger and A. parasiticus under AhCPI treatment at two different stages of development

The effect of AhCPI treatment on fungal mycelial growth was variable and depended on the stage of development in which the fungus is exposed to cystatin (Figure 2). In this study, cultures of 28 h of *A. niger* and *A. parasiticus* diminished mycelial growth with 20 and 30 μM of AhCPI, respectively (Figures 2A and B). Interestingly, *A. niger* exhibited a dramatic decrease in mycelial growth compared with *A. parasiticus* (Figures 2A and B). On the contrary, when AhCPI was added to cultures of 43 h, mycelial growth was slightly inhibited (Figures 2C and D). When the cystatin exposure time (29 h) is the same in both cultures (at 72 h in the case of cultures of 43 h and, at 57 h in the case of cultures of 28 h), the effect of AhCPI is major on younger cultures, as it is observed in Figure 2. These results indicated that AhCPI delayed mycelial growth of both fungi, but in the case of *A. niger*, its effect is major in young cultures.

In accordance with our results, AhCPI affects fungus at different levels and it greatly depends of the stage of development in which the fungus is treated. However, *A. niger* was more susceptible, since it required a lesser AhCPI concentration to inhibit spore germination compared with *A. parasiticus*. Accordingly, it has been suggested that the cystatin inhibitory effect is determined by the fungal species (Porruan et al. 2013); this hypothesis is in agreement with this study and others (Joshi et al. 1998; Pernas et al. 2000; Martínez et al. 2003; Valdés-Rodríguez et al. 2010). These results may be explained in terms of cell wall organization and composition, which are extremely variable and depend on many factors such as environmental changes, developmental stage, fungal species, etc. (Adams 2004; Free 2013).

### 3.3. Morphological changes of A. niger and A. parasiticus under AhCPI treatment by microscopic observations

**Figure 4.**
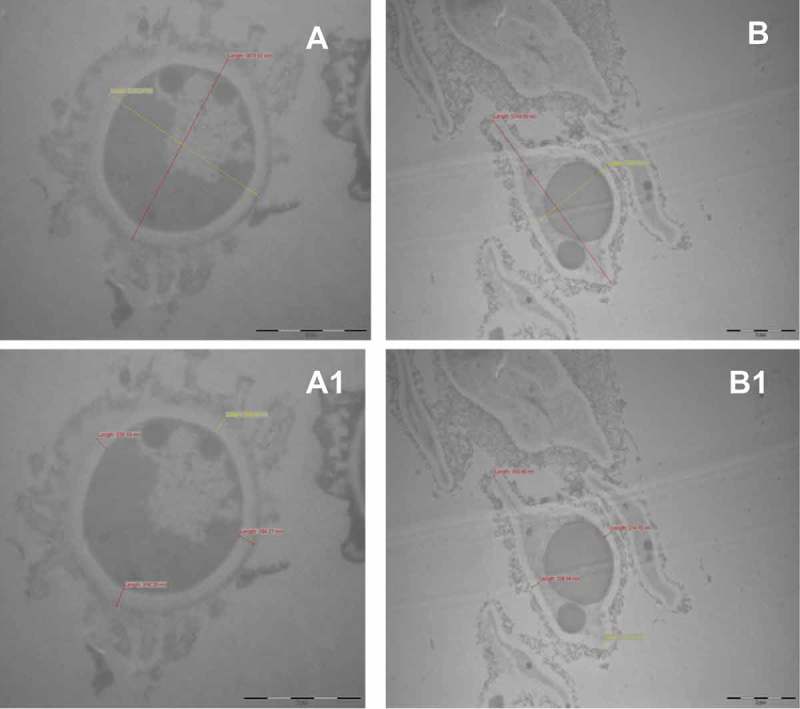
Electron microscopic images showing the effect of cystatin on spore morphology of *Aspergillus parasiticus* (**A**) Untreated spores of *A. parasiticus* growing in PDB; picture shows line length of distal points; the calculation of aspect ratio was obtained by dividing the length by the width of spores (3670.52/3275.04 nm) and **A1** shows cell wall thickness of various points (236.78; 268.58; 294.27 and 316.26 nm). Bar = 2 μm. (**B**) Spores of *A. parasiticus* treated with 30 μM AhCPI, aspect ratio (5749.36/2765.76 nm); and (**B1**) Cell wall thickness size of various points (168.48; 210.61; 214.78; 236.04). Magnification bar = 2 μM. Numeric data was obtained with iTEM-FEI program.

Light microscopic observations showed that AhCPI affected at level of spore germination (Figure 3); the addition of 20 μM inhibited completely spore germination of *A. niger* (Figure 3A panel 3); while in *A. parasiticus*, at the same concentration of AhCPI, some germlings were presented; however, no mycelial growth was observed (Figure 3B panel 3). Electron microscope images showed that AhCPI affects cell integrity. Untreated spores of *A. parasiticus* presented a thick pigmented shell on the cell wall (Figure 4 panels A and A1), which was not presented in spores treated with cystatin (Figure 4 panels B and B1). After treatment with AhCPI, spores of *A. parasiticus* presented an amorphous and unornamented layer at the surface. In addition, parameters such as aspect ratio and thickness of cell wall were compared with those of untreated spores. The aspect ratio of untreated spores of *A. parasiticus* was 1.1 with a nearly spherical shape; however, cystatin-treated spores presented an aspect ratio of 2 with an ameboide shape. An effect in cell wall thickness by cystatin treatment also was observed, untreated spores showed range between 236.78 and 316.26 nm while, after treatment with AhCPI spores presented a range between 168.48 and 236.04 nm. Furthermore, cystatin treatment induced internal disorganization as well as an increase in vacuole size.

**Figure 5.**
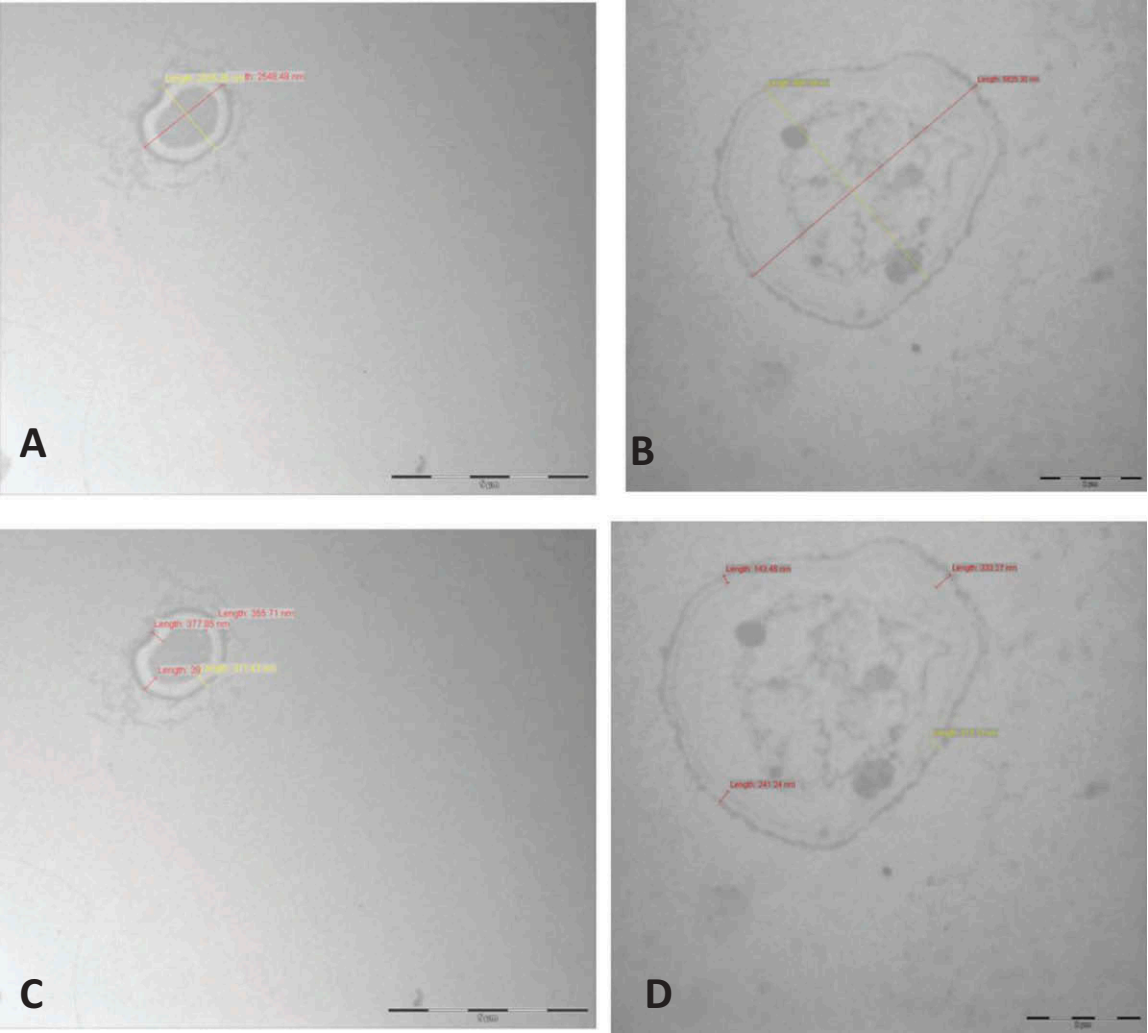
Electron microscope images showing the effect of cystatin on spore morphology of *Aspergillus niger*. (**A**) Untreated spores of *A. niger* growing in PDB, picture shows line length used to calculate the aspect ratio (2548.48 /2035.35) and (**C**) shows cell wall size of various points (311.43; 355.71; 377.85; 390.04). Magnification bar = 5 μM. (**B**) Spores of *A. niger* treated with 20 μM AhCPI, aspect ratio (5825.30/4891.59 nm); and (**D**) cell wall thickness of various points (143.46; 213.13; 241.24; 333.27). Scale bar = 2 μM. Numeric data was obtained with iTEM-FEI program.

Interestingly, the aspect ratio of *A. niger* untreated spores was 1.2 whereas spores after treatment with cystatin presented an aspect ratio of 1.19 (Figure 5, panels A and A1). Cell wall thickness of untreated spores ranged between 311.43 and 390.04 nm and, after treatment with AhCPI, cell wall thickness ranged between 143.46 and 333.26 nm (Figure 5 panels B and B1). Other morphological changes exhibited by *A. niger*, such as cytoplasm density alterations, deformation and visible structural disorganization, were observed and the cytoplasm of treated spores contained transparent material instead of the dark material presented in untreated spores. Indeed, disruption of function or structure of the fungal cell wall is one of the main mechanisms of many types of antifungal compounds (Hector 1993; George and Selitrennikoff 2006; Aderiye and Oluwole 2015). In opposition, evidence from Hv-CPI showed that the antifungal effect of this cystatin and its derived mutants does not correlate with their activities as proteinase inhibitors. Recombinant Hv-CPIs affected in their activities as proteinase inhibitors due to point mutations (R38 → G, Q63→L, and Q63→P) conserved its antifungal properties (Martínez et al. 2003; Cheng et al. 2014). In the same way, no correlation was found between the inhibition of papain activity and the antifungal effect of different segments of the tarocystatins (Wang et al. 2008). It has been shown that the N-terminal region of tarocystatin had a major antifungal activity that the complete protein, whereas the C-terminal segment of the protein did not present any antifungal properties (Wang et al. 2008). Additionally, it has been proved that SAP16, a trypsin inhibitor isolated from *Helianthus annuus* inhibited ascospore germination, reduced mycelial growth and caused modifications in fungal membrane permeability of *Sclerotinia scleroticum*; these effects were not related with inhibition of any fungal protease (Giudici et al. 2000). However, AhCPI concentration required to arrest the fungal proteolytic activity was higher compared to the concentration used to inhibit fungal growth (Valdés-Rodríguez et al. 2010), suggesting that the antifungal activity of AhCPI is probably not mediated by the inhibition of fungal proteinases.

### 3.4. Stress response of spores treated with cystatin

**Figure 6.**
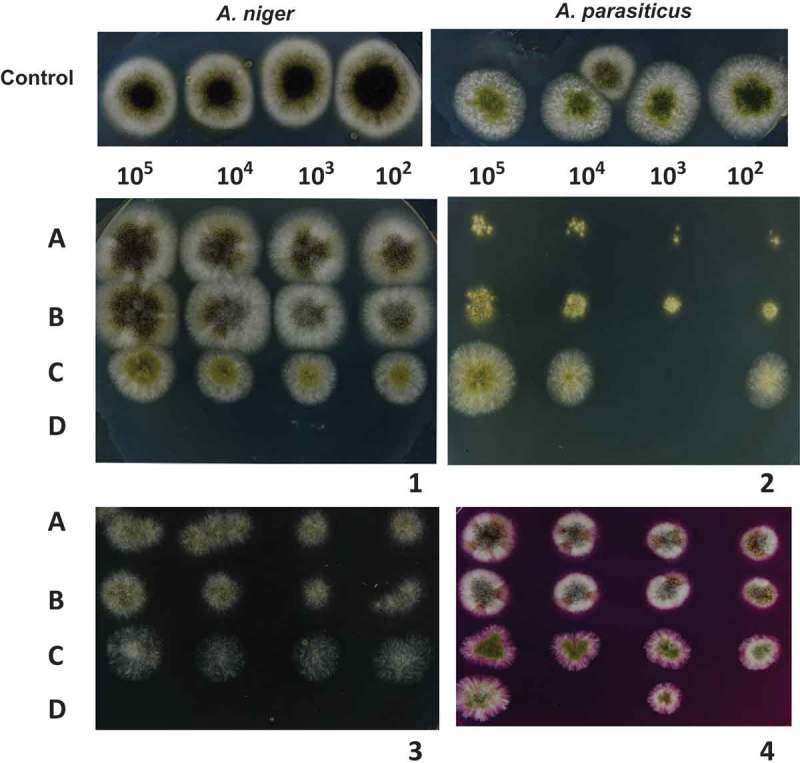
Stress response of fungi after AhCPI treatments. A control of untreated spores of both fungi growing in PDA without stressful conditions is shown. (**A**) *A. niger* without AhCPI; (**B**) Spores of *A. niger* treated with 15 μM AhCPI; (**C**) spores of *A. parasiticus* without AhCPI; and (**D**) Spores of *A. parasiticus* treated with 20 μM AhCPI. Plates were incubated for 72 h in PDA containing (**1**) 1% H_2_O_2_; (**2**) 2 mM LiCl; (**3**) 2 M Sorbitol; and (**4**) 1% Rose bengal.

The electron microscopic evidence presented in this study strongly suggests that AhCPI antifungal activity over *A. niger* and *A. parasiticus* could be given by an alteration on cell integrity. Therefore, the stress response of these fungi might be affected. To analyse this hypothesis, treated spores were subjected to different stress assays: osmotic, oxidative and ionic stress (Figure 6). *Aspergillus niger* showed no difference in stress sensitivity compared with spores treated with AhCPI (Figures 6A and B). On the contrary, *A. parasiticus* spores presented high susceptibility to all the stresses, indicating that AhCPI affected their response to oxidative stress caused by H_2_O_2_ and rose bengal, osmotic stress generated by sorbitol and ionic stress caused by LiCl (Figures 7B and C). At this point, it is important to mention that the filamentous fungus *A. niger* is highly resistant to osmotic and ionic stresses (Wucherpfennig et al. 2011).

## 4. Conclusion

These results confirmed the antifungal activity of AhCPI against the mycotoxigenic fungus *A. parasiticus*, and the GRAS fungus *A. niger* by inhibiting spore germination and mycelial growth. Additionally, this study showed some evidence that AhCPI provokes impaired fungal cell integrity, and in *A. parasiticus*, sensitivity to stressful conditions.

## Disclosure statement

No potential conflict of interest was reported by the authors.
